# Comparison of Enzyme-Linked Immunosorbent Assay, Surface Plasmon Resonance and Biolayer Interferometry for Screening of Deoxynivalenol in Wheat and Wheat Dust

**DOI:** 10.3390/toxins8040103

**Published:** 2016-04-11

**Authors:** Melanie Sanders, Daniel McPartlin, Kara Moran, Yirong Guo, Mia Eeckhout, Richard O’Kennedy, Sarah De Saeger, Chris Maragos

**Affiliations:** 1 Mycotoxin Prevention and Applied Microbiology Research Unit, United States Department of Agriculture-Agricultural Research Service-National Center for Agricultural Utilization Research (USDA-ARS-NCAUR), 1815 N. University Street, Peoria, IL 61604, USA; chris.maragos@ars.usda.gov; 2 School of Biotechnology, National Centre for Sensor Research and Biomedical Diagnostic Institute, Dublin City University, Dublin 9, Ireland; daniel.mcpartlin2@mail.dcu.ie (D.M.); kara.moran24@mail.dcu.ie (K.M.); richard.okennedy@dcu.ie (R.O.); 3 Institute of Pesticide and Environmental Toxicology, Zhejiang University, Hangzhou 310029, China; yirongguo@zju.edu.cn; 4 Department of Applied Biosciences, Faculty of Bioscience Engineering, Ghent University, Valentin Vaerwyckweg 1, 9000 Ghent, Belgium; mia.eeckhout@ugent.be; 5 Department of Bioanalysis, Laboratory of Food Analysis, Faculty of Pharmaceutical Sciences, Ghent University, Ottergemsesteenweg 460, 9000 Ghent, Belgium; sarah.desaeger@ugent.be

**Keywords:** deoxynivalenol, monoclonal antibodies, immunosensor, wheat, wheat dust

## Abstract

A sample preparation method was developed for the screening of deoxynivalenol (DON) in wheat and wheat dust. Extraction was carried out with water and was successful due to the polar character of DON. For detection, an enzyme-linked immunosorbent assay (ELISA) was compared to the sensor-based techniques of surface plasmon resonance (SPR) and biolayer interferometry (BLI) in terms of sensitivity, affinity and matrix effect. The matrix effects from wheat and wheat dust using SPR were too high to further use this screenings method. The preferred ELISA and BLI methods were validated according to the criteria established in Commission Regulation 519/2014/EC and Commission Decision 2002/657/EC. A small survey was executed on 16 wheat lots and their corresponding dust samples using the validated ELISA method. A linear correlation (*r* = 0.889) was found for the DON concentration in dust *versus* the DON concentration in wheat (LOD wheat: 233 μg/kg, LOD wheat dust: 458 μg/kg).

## 1. Introduction

Deoxynivalenol (DON) is a secondary metabolite produced by certain species of fungi, in particular *Fusarium graminearum* and *F. culmorum*. These fungi can infest small grains causing significant economic damage to cereal crops worldwide. Animal exposure to DON can lead to reduced food or feed consumption, abdominal distress, malaise, diarrhea, shock and death in extremely high doses [1]. To protect human and animal health, many countries have established regulatory levels for DON in grains. In the United States the advisory level is 1 mg DON/kg (1 ppm), and, in Europe, maximum levels between 200 and 1750 μg DON/kg are advised by the European Commission (European Commission Regulation (EC) No 1881/2006).

Traditional methods for identification and quantification of DON in commodities and foods consist of chromatographic methods such as high performance liquid chromatography (HPLC) and gas chromatography (GC) coupled to mass spectrometry (MS) [2,3,4,5]. Immunoassays have also been frequently used for DON detection. These include formats such as enzyme-linked immunosorbent assay (ELISA), lateral flow test strips, fluorescence polarization immunoassay (FPIA) and sensor-based immunoassays including surface plasmon resonance (SPR), flow cytometry and biolayer interferometry (BLI) [6].

Immunoassays are able to screen for DON more rapidly and with minimal sample cleanup and/or preconcentration compared to chromatographic methods. An extra advantage of sensor-based immunoassays such as SPR and BLI is that the interaction is studied in real time and without the need of labeling. Changes in refractive index are continuously detected on the surface layer of the solution in contact with the sensor surface. In BLI, the sensors are dipped into the test solution instead of having the test solution flown through small channels that can become plugged as in SPR [7,8]. Finally, the automated technology can be included in portable instruments for on-site monitoring and identification of mycotoxins [9,10,11]. Different SPR-based immunoassay methods for DON determination in wheat cereals are previously described. All of them consist of an organic sample preparation followed by a fast quantitative determination and give promising results even for multi-mycotoxin analysis [12,13,14]. The use of BLI for mycotoxin screening is still limited [15].

As mentioned in Sanders *et al.*, DON accumulates in wheat dust. Former research highlighted the risk of developing respiratory diseases with people working in the agricultural sector, especially during the process of grain harvesting and threshing [16,17]. Dust contaminated with mold spores and/or mycotoxins may cause asthma, allergic alveolitis, chronic bronchitis, allergic rhinitis, mucous membrane irritation, certain infectious diseases and cancer due to long-term inhalation [18,19,20].

The American Conference of Governmental Industrial Hygienists (ACGIH, 1997) has defined three grain dust mass fractions in relation to potential health effects: (1) the inhalable fraction (median aerodynamic diameter of 100 μm that enters the airways region); (2) the thoracic fraction (median aerodynamic diameter of 10 μm which deposits in the tracheobronchial regions) and (3) the respirable fraction (median aerodynamic diameter of 4 μm which enters the gas-exchange regions) [21]. Thus, the smaller the dust particle size and the bigger the surface area, the more dangerous in terms of health risks. As indicated in Sanders *et al.*, the most abundant dust fraction in wheat has a particle size smaller than 50 μm. The health risk should surely be taken seriously [16].

There is a more homogenous distribution of mycotoxins in grain dust compared to grain cereals. Due to the possibility of continuously sampling through the entire grain movement process (recovery of only 20 g dust out of a wheat bulk lot of 100 kg), a more representative sample can be taken [16]. For dust, a grinding step is not necessary prior to sample extraction making it less time-consuming than the conventional cereal sampling. At the moment, no screening technique exists for the determination of mycotoxins in grain dust as no regulatory limits for mycotoxins in grain dust and no mycotoxin absorption data after inhalation are yet available.

The objectives of the current research were to develop an aqueous sample preparation for wheat and wheat dust and to couple this sample preparation to ELISA, SPR and BLI to compare their suitability for DON detection in cereal matrix. In addition, the suitability of using wheat dust instead of wheat grain for on-site screening was further investigated.

## 2. Results and Discussion

### 2.1. Chemical Composition of Wheat Dust

The water plus organic fraction as determined by the difference in weight before and after incineration resulted in 27.94% and 11.65% for the two different wheat dust batches, which lies within the range mentioned by Martin (1981) [22]. The mineral composition of the inorganic fraction is shown in Table 1. In batch 1, the most predominant minerals were Fe, K and P, and, for batch 2, most mineral components were present in a higher quantity with extremely high values for Fe, K, Mg, Na and P. Therefore, it can be concluded that the water and organic fraction and the mineral composition of wheat dust can be different from batch to batch.

It is generally known that changing the composition of phosphate buffered saline (PBS) or PBS Tween 20 (PBST) by adding extra sodium or phosphate can have an influence on the outcome of an immunoassay. Therefore, it can be concluded that a different mineral composition of wheat dust may change the immunoassay results in the same way.

### 2.2. Optimization of Sample Preparation

The ELISA results using wheat dust extracts without filtration are presented in Table 2. As seen for the 4000 μg/kg spike, the best recoveries were found with the 1/100 diluted samples. This is normal as the linear range of the 10H10 antibody in wheat dust matrix lays in between 110 μg/kg and 4060 μg/kg, taking into account the 0.5 g of wheat dust and 5 mL of extraction solvent. Based on this linear range, only the 1/10 and 1/100 dilution would be quantifiable. No significant difference was seen between the use of filter and no filter (*p*-value = 0.378), so it was clear that small particles did not influence the antibody or the ELISA format. Therefore, no filter was used in further experiments. A very low recovery (below 32%) was observed for the spiked wheat dust samples at a level of 12,000 μg/kg and 20,000 μg/kg. Therefore it was concluded that a higher extraction volume or a certain percentage of methanol was necessary to extract large DON concentrations.

No difference in recovery was observed between the use of water and water with 10% methanol as extraction solvent (*p*-value = 0.442) with a maximum recovery of 52%. When increasing the extraction volume of water up to 10 mL, 25 mL and 50 mL, the recovery increased with a maximum and optimal recovery value at 50 mL extraction volume and no dilution of supernatant or 25 mL extraction volume and a dilution of supernatant in PBS (1:1, *v*/*v*) prior to ELISA. Using the optimized extraction volume, recovery values at spiking levels of 4000 μg/kg, 8000 μg/kg, 12,000 μg/kg, 16,000 μg/kg and 30,000 μg/kg lay in the range 75%–153%. The extraction duration was the last parameter which was optimized. Spiked dust samples were extracted for 10, 20, 30 min and 1 h and the recovery was calculated and compared. A maximum recovery was reached after 30 min of water extraction. 

For wheat extracts, the influence of centrifugation (3000 *g*, 5 min) prior to ELISA was determined and no difference in signal intensity was seen compared to the non-centrifuged ones (*p*-value = 0.098). As no difference in recovery was noticed between an extraction time of 30 min and 1 h (*p*-value = 0.488), it was decided to use the shorter extraction time of 30 min. Using 30 mL extraction solvent, mean recovery results of 100.17% were measured, which was an increase in recovery of 20% compared to 5 and 10 mL extraction solvent. Similar recovery results were obtained when using 15 mL extraction solvent and a dilution in PBS (1:1, *v*/*v*) before ELISA.

A better repeatability in relative ELISA signal intensities was seen using a dilution of the extract in PBS (1:1, *v*/*v*). Therefore, wheat and wheat dust samples were further extracted using 15 mL and 25 mL water and subsequent dilution in PBS before analysis.

The optimized sample preparation conditions include an extraction volume of 15 mL or 25 mL of water followed by overhead shaking for 30 min. A 1:1 (*v*/*v*) dilution of the non-filtered supernatant solution in the corresponding buffer was chosen prior to analysis.

### 2.3. Optimization of Immunoassay Conditions

ELISA conditions were previously optimized [23] and are described in Materials and Methods.

During an SPR preconcentration study, 25 μg/mL and 100 μg/mL DON-ovalbumin (DON-OVA) diluted in 10 mM sodium acetate pH ranging from 3.4 to 4.4, were injected over the sensor surface to check for maximal signal (RU). As a maximum signal was determined with 100 μg/mL DON-OVA in 10 mM sodium acetate pH 4.2, this was used for coating of a sensor chip. A total amount of 12,000 RU of DON-OVA remained after coating and blocking of the CM5 sensor surface. The interaction between antibody and immobilized DON-OVA on the sensor chip was determined by monitoring real-time RU changes for a range of antibody concentrations (31.25 ng/mL, 62.5 ng/mL, 125 ng/mL, 250 ng/mL and 500 ng/mL) in 0.01 M 2-[4-(2-hydroxyethyl)piperazin-1-yl]ethanesulfonic acid (HEPES) containing 0.15 M sodium chloride pH 7.4 (HBS-N^+^ ). The injection of the highest antibody concentration (500 ng/mL) gave an increase in signal of 100 RU. The sensorgram of the antibody was compared to the Langmuir model and no significant difference was observed between the resulting curve and the model curve (χ^2^ = 0.489). Also, different regeneration buffers were evaluated, namely guanidine HCl 6 M in glycine 50 mM pH 2.0 and 10 mM NaOH [7]. The 10 mM NaOH was further used as the guanidine seemed to strip the DON-OVA off the sensor surface.

In BLI, the interaction between the antibody and DON-OVA was determined in real-time by observing response changes for different antibody concentrations (2.5 μg/mL, 5 μg/mL, 10 μg/mL, 25 μg/mL, 40 μg/mL and 50 μg/mL) in C-PBS. As an optimum maximum response was seen for 5 μg/mL antibody, this concentration was used for the DON competitive study. No significant difference was seen between the antibody sensorgram and its Langmuir model with a χ^2^ value of 0.256.

### 2.4. Comparison between ELISA and Sensor-Based Immunoassays

**Figure 1:**
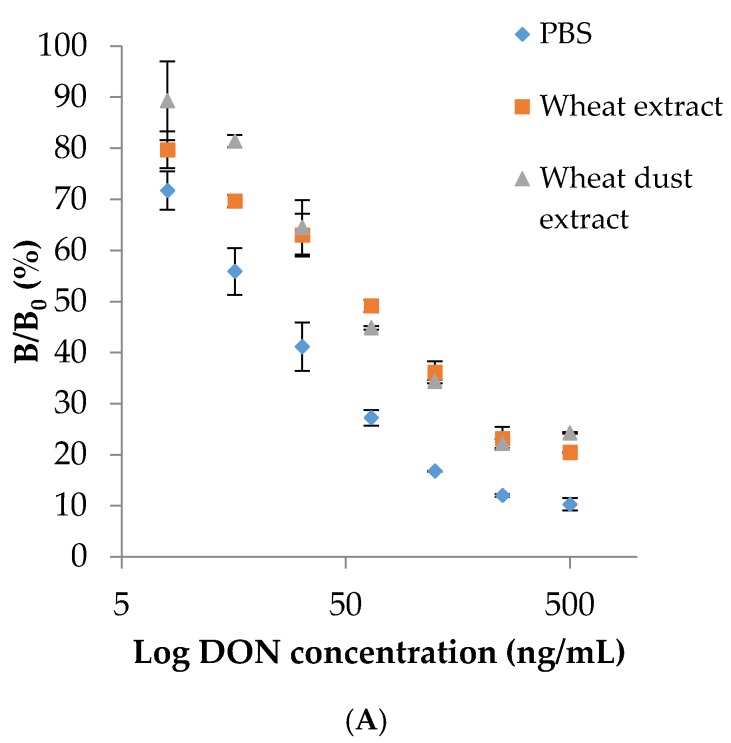

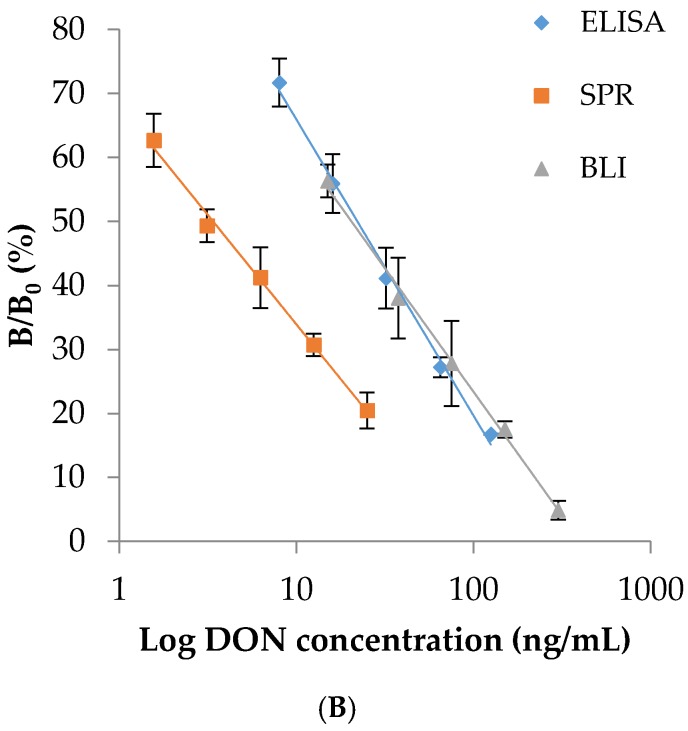
(**A**) effect of wheat and wheat dust matrices on the direct ELISA for DON; (**B**) comparison of three immunoassay formats (direct ELISA, indirect SPR and indirect BLI) as indicated by the linear calibration ranges in buffer.

As described in Sanders *et al.*, the highest sensitivity (determined by the half maximal inhibitory concentration (IC_50_ value)) of the DON monoclonal antibody was obtained using direct ELISA compared to indirect ELISA [23]. Therefore, the direct ELISA format was further used for the development of a wheat and wheat dust screening method for DON. In direct ELISA, an IC_50_ value of 22 ng/mL was determined in PBS. With wheat and wheat dust extracts, this IC_50_ value increased until respectively 56 ng/mL and 61 ng/mL, as illustrated in Figure 1A.

The direct assay format did not work for SPR and BLI, as the DON conjugates did not cause significant changes in refractive index. In these sensor-based immunoassays, the indirect format with DON-OVA coated sensors was used. In SPR, an IC_50_ value of 3.17 ng/mL for DON was determined. When injecting blank wheat and wheat dust extract over the sensor surface, the signal decreased with 3000 RU. In addition, no signal decrease was observed with DON spiked wheat and wheat dust extracts. Thus, the matrix effect made it impossible to analyze aqueous wheat and wheat dust extracts with SPR. This is in contrast to the successful published SPR methods using organic sample preparation procedures [12,13,14].

For BLI, IC_50_ values of 18.2 ng/mL, 75.1 ng/mL and 76.4 ng/mL for DON were determined in C-PBS, wheat and wheat dust extract. The χ^2^ values for wheat (0.228) and wheat dust (0.175) revealed no significant differences between the antibody sensorgrams and their Langmuir models. The differences in IC_50_ values between direct ELISA, SPR and BLI in buffer are illustrated in Figure 1B.

To further compare the use of ELISA and BLI, the affinity of the antibody towards the DON-OVA was measured in both immunoassays. The equilibrium dissociation constant (K_D_) was determined as this is inversely related to the affinity. The K_D_ determines the concentration of antibody needed for a particular experiment and so the lower the K_D_, the higher the affinity of the antibody. Using the non-competitive ELISA format, K_D_ values of 7.7 × 10^−11^ M (PBS), 7.7 × 10^−11^ M (wheat) and 8.9 × 10^−11^ M (wheat dust) were measured. The BLI determined K_D_ values were 8.5 × 10^−14^ M (C-PBS), 3.5 × 10^−12^ M (wheat) and 3.2 × 10^−12^ M (wheat dust). This means that the DON-OVA conjugate can be detected in a picomolar (pM) range in ELISA and in a range at least 20 times lower in BLI. In BLI, the K_D_ values also seem to be influenced by the wheat and wheat dust matrix. Furthermore, the higher the affinity of the antibody to DON-OVA, the lower the sensitivity of the antibody. This means that the antibody would show a lower sensitivity in BLI, than in ELISA. This statement was not proven by the determined IC_50_ values. To compare the antibody affinity for DON-OVA and DON, competitive ELISA experiments were performed in PBS. In competitive ELISA, a K_D_ value of 5.0 × 10^−9^ M was determined, which means that the antibody shows a 100 times higher affinity for DON-OVA than for DON.

BLI gives a higher antibody affinity than indirect ELISA. Still, ELISA can determine DON contaminations in a very low range and is for now considered as the method of choice for on-site screening. Therefore, both methods were validated for the determination of DON in wheat and wheat dust matrix.

### 2.5. Method Validation

The ELISA and BLI methods were successfully validated for DON in wheat and wheat dust based on European Commission Regulation 519/2014/EC and Commission Decision 2002/657/EC. A DON spiking range from 100 to 3000 μg/kg and 500 to 15,000 μg/kg was taken for the wheat and wheat dust calibration curve, respectively. The linearity was evaluated by the correlation coefficient (*r*) and a lack of fit test.

For the determination of the fitness for purpose of the screening method, the STC for wheat was set at the maximum DON regulatory level in unprocessed cereals other than durum wheat, oats and maize (1250 μg/kg) and the contamination level of the 20 positive control samples was taken around this STC, namely at 1500 μg/kg. As for DON in wheat dust, no maximum levels are yet established, the STC was chosen at a higher concentration than for wheat based on the research results described in Sanders *et al.* (2013) and was set at 8000 μg/kg [16]. The calculations of the cut-off level and the rate of false suspected results were based on the relative responses of the blank samples and positive control samples. For the determination of the false suspect results, a *t*-value of 5.68 and 7.32 was calculated for respectively wheat and wheat dust in ELISA. For BLI, *t*-values of 2.55 and 4.68 were determined for respectively wheat and wheat dust. This value corresponds to the event that a result of a negative control sample is above the cut-off value. Based on this *t*-value and the degrees of freedom (19), a probability of false suspect samples for a one tailed distribution of less than 0.01% was determined for both wheat and wheat dust in ELISA and BLI. The apparent recovery, inter- and intraday precision were measured at seven different concentration levels; however, only the data obtained at medium and high levels were recorded. An overview of the validation results is given in Table 3.

Based on the described validation results, both ELISA and BLI methods for wheat and wheat dust were shown to be acceptable for their purpose.

### 2.6. Analysis of Wheat and Wheat Dust Samples

The DON content of naturally contaminated wheat and dust samples (*n* = 16) was determined according to the described direct ELISA method. As ELISA and BLI showed similar characteristics, the samples were only analyzed with one method. ELISA was chosen because of its possibility to use for on-site screening.

Each wheat and corresponding wheat dust sample was analyzed once. All wheat samples were contaminated with DON up to 1113 μg/kg with a mean contamination level of 244 μg/kg (median = 75 μg/kg). Dust samples clearly showed higher levels in a range from 607 μg/kg to 14,043 μg/kg with a mean contamination of 5012 μg/kg (median = 1518 μg/kg). According to the Commission Regulation (EC) No 1881/2006 of 19 December 2006 setting maximum levels for certain contaminants in foodstuffs, for DON in unprocessed cereals other than durum wheat, oats and maize the maximum limit was set at 1250 μg/kg. No wheat samples exceeded this maximum limit.

### 2.7. Dust Correlation Study

**Figure 2:**
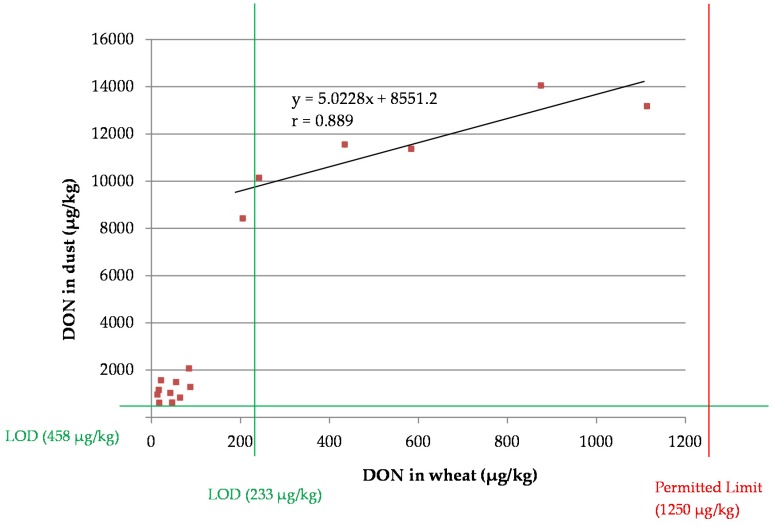
A scatterplot of the deoxynivalenol (DON) concentration in dust (*y*-axis) *versus* wheat (*x*-axis). A linear correlation between the DON concentration in wheat dust *versus* the DON concentration in wheat was observed (*r* = 0.889), when not considering the data points with a DON concentration in wheat lower than 200 μg/kg (< limit of detection (LOD)).

The ELISA results for each wheat sample and the corresponding dust sample are presented in a scatterplot (Figure 2). When not considering the data points with a DON concentration in wheat lower than 200 μg/kg (<limit of detection (LOD)), a statistically significant linear correlation (*r* = 0.889, *p*-value = 0.044) was found. The slope of the trend line described a value of 5.023, which corresponds to a fivefold accumulation of DON on small particles and is different to the slope (13.192) found after liquid chromatography tandem mass spectrometry (LC-MS/MS) analysis of wheat and their corresponding wheat dust samples (Sanders *et al.* (2013)) [16]. As limited wheat and corresponding wheat dust samples were obtained and only at a concentration level in wheat lower than 1200 μg/kg, the uncertainty of the slope corresponding to the linear curve is high.

## 3. Materials and Methods

### 3.1. Reagents and Chemicals

DON standard was obtained from Fermentek (Jerusalem, Israel). DON-ovalbumin (DON-OVA) and DON-horseradish peroxidase (DON-HRP) were synthesized by the *N*,*N*′-carbonyldiimidazole (CDI) coupling reaction, adopted from published literature [24,25]. The 10H10 monoclonal DON antibody was previously developed and characterized. It was considered a broad specific antibody showing cross reactivity against its acetylated forms 3-acetyl-DON (147%) and 15-acetyl-DON (65%) [23,26].

Colorburst™ blue 3,3′,5,5′-tetramethylbenzidine (TMB) substrate solution containing hydrogen peroxide was supplied by Alerchek (Springvale, ME, USA). Rabbit anti-mouse immunoglobulin G (anti-mouse IgG secondary antibody; protein concentration of 2.5 g/L) was purchased from DakoCytomation (Glostrup, Denmark). Rabbit anti-mouse IgG secondary antibody labeled with HRP was obtained from Sigma-Aldrich (Bornem, Belgium).

Several buffers were used in the immunoassays. These include 10 mmol/L phosphate buffered saline (PBS: 0.01 M disodium phosphate, 1.8 mM potassium phosphate, 2.7 mM potassium chloride and 0.14 M sodium chloride, adjusted to pH 7.2), PBS Tween 20 (PBST: PBS containing 0.05% Tween 20 (*v*/*v*)), carbonate buffered saline (CBS: 0.07 M sodium bicarbonate, 0.03 M sodium carbonate, pH 9.6), HBS-N^+^ buffer containing 0.01 M 2-[4-(2-hydroxyethyl)piperazin-1-yl]ethanesulfonic acid (HEPES) pH 7.4 and 0.15 M sodium chloride, 50 mM phosphate buffer (PB: 0.035 M disodium phosphate, 0.015 M sodium phosphate, adjusted to pH 7), casein-PBS (C-PBS: 0.1% casein in PBS), Glycine/*N*,*N*-dimethylformamide (Gly/DMF) buffer consisted of 0.1 M glycine (pH 3) mixed with DMF (4:1, *v*/*v*).

Water was obtained from a Milli-Q SP Reagent water system from Millipore Corp. (Brussels, Belgium). Other chemicals and solvents were of reagent grade or better and purchased from major suppliers.

### 3.2. Wheat Dust Collection and Chemical Composition

Blank wheat and 16 different naturally contaminated wheat samples were randomly collected in Belgium and Hungary in the framework of the European FP7 MycoHunt project (“Rapid biosensor for the detection of mycotoxin in wheat”). As only 20 g of dust can be recovered out of a wheat bulk lot of 100 kg, extensive efforts were made to receive a sufficient amount of DON contaminated wheat samples from grain millers, but only 16 samples could be obtained. Every wheat lot was mixed in a vertical screw mixer and one part of each wheat sample was ground using the M20 grinder (Ika Werke, Staufen, Germany). Out of the remaining part of the wheat sample, dust was aspirated by the use of the dust collection facility described at Sanders *et al.* [16].

The chemical composition of two different batches of wheat dust was determined to better understand possible interfering components for the monoclonal antibody within the assay. First, between 3 and 5 g of wheat dust was weighed in a cup and placed in an oven. Starting from room temperature, every 5 h, the temperature of the oven increased with 5 °C until a temperature of 550 °C was reached. Afterwards, the cup was cooled down in a desiccator and the water together with the organic fraction was determined by calculating the difference in weight before and after incineration. Based on Dashek *et al.* (1986), the moisture content of spring wheat dust has a value between 4.97% and 8.08% depending on the duration of drying at 60 °C [27]. Previous researchers have measured a percentage of ash between 7.9% and 28.5% [22,28]. Then, 100 mg of the ash was redissolved in 5 mL 6 M HCl followed by 5 mL 3 M HCl while heating. After cooling down, 50 mL of water was added and the solution was filtered by the use of a Whatman No 5 filter (VWR International, Zaventem, Belgium). The filtered solution was analyzed by inductively coupled plasma with atomic emission spectroscopy (ICP-AES) to determine and quantify the mineral composition of the inorganic fraction of the wheat dust.

### 3.3. Sample Reparation

One gram of wheat or half a gram of the corresponding wheat dust was weighed in a Gosselinextraction tube (50 mL). Water was chosen as extraction solvent, because of its ease of use on-site and because of its suitability with antibodies.

During the sample preparation optimization, blank wheat and wheat dust extracts were spiked with DON standard dissolved in acetonitrile (100 ng/μL) at a level of 3000 μg/kg for wheat and between 4000 μg/kg and 30,000 μg/kg for wheat dust. Extraction was performed using the Agitator decanter overhead shaker (Agitelec, J. Toulemonde and Cie, Paris, France). The best sample preparation conditions were determined based on the measured apparent recovery values in ELISA.

First of all, half a gram of wheat dust was spiked with DON at levels of 4000 μg/kg, 12,000 μg/kg and 20,000 μg/kg and extracted by overhead shaking for 1 h using 5 mL of water. After centrifugation, half of the supernatant was filtered using a Whatman No 4 filter (VWR International, Zaventem, Belgium) and diluted 1/10, 1/100 and 1/1000 with water prior to direct ELISA. The experiment was repeated using only wheat dust samples spiked at 20,000 μg/kg extracting with water or water with 10% methanol (5 mL) to evaluate the influence of organic solvent on the extraction efficiency. Other parameters that were evaluated were the extraction volume (5 mL, 10 mL, 25 mL and 50 mL) and extraction duration (10 min, 20 min, 30 min and 1 h). For the optimization of wheat extraction, wheat was spiked with DON at a level of 3000 μg/kg and extracted by overhead shaking for 30 min or 1 h using 5 mL, 10 mL or 30 mL of water.

In the optimized sample preparation procedure, the wheat and wheat dust extraction was performed by adding 15 mL or 25 mL of water for 30 min. This was followed by a short centrifugation at 3000 *g* for 5 min to remove small particles from the supernatant. The supernatant solution was diluted 1:1 (*v*/*v*) in PBS (ELISA), HBS-N^+^ buffer (SPR) or 0.1% casein (BLI) prior to analysis.

### 3.4. Analysis

#### 3.4.1. ELISA

Competitive direct ELISA was used to characterize the anti-DON monoclonal antibody. All incubations, except for the first coating step, were carried out at 37 °C, and after each incubation, the plates were washed three times (300 μL/well) with PBST using an automatic microplate washer “96 PW” (TECAN, Salzburg, Austria). High-binding polystyrene 96-well microplates were coated with 100 μL/well of anti-mouse IgG secondary antibody diluted in CBS (5 μg/mL). After overnight incubation at 4 °C, the plates were blocked with 2% (*w*/*v*) skim milk in PBS (300 μL/well) for 30 min. An appropriate dilution of the 10H10 monoclonal antibody (100 μL/well) was added to the 96-well plate, followed by an incubation step of 1 h. After this, standard solutions of DON and PBS control were added (50 μL/well) for the standard curve and wheat or wheat dust extract (50 μL/well) for the unknown grain sample. DON-HRP (50 μL/well) was added for competition of binding to the antibody and this was incubated for another hour. Then, 100 μL/well of TMB substrate solution was added. The reaction was stopped after 15 min with 2 M sulphuric acid (50 μL/well), and the absorbance at 450 nm was measured by a Bio-Rad model 550 microplate reader (Richmond, CA, USA).

Standard competitive curves were obtained by plotting relative absorbance (ratio of absorbance measured at the standard concentration and zero concentration: *B*/*B*_0_ × 100%) against the logarithm of analyte concentration. Half maximal inhibitory concentration (IC_50_) values were determined to assess the assay sensitivity. Matrix-matched standard competitive curves were made by spiking blank wheat and wheat dust samples to obtain DON concentration levels of 3000 μg/kg and 15,000 μg/kg, respectively. Blank samples were considered as blank if no DON contamination was found after LC-MS/MS analysis (LOD 8.5 μg/kg for wheat and 358 μg/kg for wheat dust).

The affinity of the monoclonal antibody against DON and DON-OVA was evaluated using competitive and non-competitive indirect ELISAs [29,30]. For competitive indirect ELISA different DON concentrations (0, 10, 20, 40, 80, 160, 320, 640 ng/mL) were incubated at room temperature with 133 ng/mL of the monoclonal antibody. After 2 h, the mixture was added to a DON-OVA (100 ng/mL) coated 96-well microplate. During non-competitive indirect ELISA, the monoclonal antibody (40, 66.5, 80, 133 ng/mL) was added to a microplate coated with different DON-OVA concentrations (12.5, 25, 50, 100 ng/mL). After 1 h incubation at 37 °C, IgG secondary antibody labeled with HRP (1/5000, *v*/*v*) was added and this was incubated for another hour. Then, 100 μL/well of TMB substrate solution was added and the absorbance was determined as for direct ELISA.

#### 3.4.2. Surface Plasmon Resonance

For SPR analysis, a Biacore 3000 (GE Healthcare, Uppsala, Sweden) SPR instrument was used. DON-OVA was immobilized on a CM5 sensor chip (GE Healthcare, Uppsala, Sweden) by the use of amine coupling. During the immobilization, HBS-N^+^ buffer was used as a running buffer at a flow rate of 10 μL/min. The sensor chip was activated using a mixture of 400 mM 1-ethyl-3-[3-dimethylaminopropyl] carbodiimide (EDC) and 100 mM *N*-hydroxysuccinimide (NHS) in water. Then, DON-OVA diluted to 100 μg/mL with 10 mM sodium acetate (pH 4.2), was loaded on the activated sensor chip. Finally, a 1 M ethanolamine-HCl solution (pH 8.5) was used as a blocking solution. The activation, immobilization and blocking was carried out for 2547 s. Then, the DON antibody (500 ng/mL) was injected over the surface at 10 μL/min for 200 s, followed by a dissociation phase which consisted of a flow of HBS-N^+^ buffer (30 μL/min) for 700 s. After the dissociation, the surface was regenerated by the injection of 10 mM NaOH at 10 μL/min for 60 s.

To determine the assay sensitivity, different DON concentrations in HBS-N^+^ buffer or wheat (dust) extract were mixed with 500 ng/mL of monoclonal antibody and injected over the sensor surface. Calibration curves were obtained and IC_50_ values were calculated from the response units (RU) at the midpoint of the calibration curve. For the determination of the affinity of the monoclonal antibody towards the DON-OVA coated on the sensor chip, different concentrations of the antibody (31.25 ng/mL, 62 ng/mL, 125 ng/mL, 250 ng/mL and 500 ng/mL) were passed over the sensor surface.

The BIAevaluation software (GE Healthcare, Uppsala, Sweden) was used for the calculations, using a 1:1 Langmuir model and local fitting of the data.

#### 3.4.3. Biolayer Interferometry

BLI experiments were conducted using an Octet Red and aminopropylsilane (APS) sensors, manufactured by ForteBio (Menlo Park, CA, USA). The experiments were performed using the method described by Maragos [15]. Briefly, DON-OVA was immobilized on the sensor tips by incubating the sensors in 0.2 mL diluted conjugate (25 μg/mL in PB) overnight at ambient temperature. To reduce the impact of non-specific binding to the sensor surface, the sensors were further blocked by incubating them in a solution of 1% casein in PBS for a minimum of 2 h at ambient temperature. Then, the coated and blocked sensors were incubated for 3 min in 15% sucrose (*w*/*v*) and equilibrated with C-PBS before use. The regeneration of the sensors was performed with Gly/DMF. All experiments consisted of repeated cycles, each having four steps: incubation with C-PBS to establish a baseline signal (equilibration) for 60 s, incubation with the test mixture containing anti-DON monoclonal antibody (association) for 180 s, incubation with C-PBS (dissociation) for 120 s and incubation with regeneration buffer to remove the antibody in preparation for the next cycle (regeneration) for 90 s. All experiments were conducted at 30 °C and in a total volume of 0.2 mL of test solution.

The affinity and sensitivity of the monoclonal antibody to the DON-OVA sensor surface was determined using 5 μg/mL of the antibody solution and different DON concentrations in C-PBS or wheat (dust) extract. Calibration curves were set up using the BLI response as a function of the DON concentration.

Data were analyzed with the Octet Data Analysis Software 6.3 (ForteBio, Menlo Park, CA, USA), using a 1:1 Langmuir model and local fitting.

An overview of the different immunoassay formats used for the determination of antibody sensitivity (IC_50_ value) and affinity is given in Table 4.

### 3.5. Method Validation

The ELISA and BLI methods for the determination of DON in wheat and wheat dust were validated according to the criteria for the validation of semi-quantitative screening methods described in European Commission Regulation 519/2014/EC and Commission Decision 2002/657/EC. Twenty blank wheat and wheat dust samples and twenty wheat and wheat dust samples spiked at the screening target concentration (STC) (1500 μg/kg for wheat and 8000 μg/kg for wheat dust) were analyzed over 5 different days. The DON concentration was determined using matrix-matched standard competitive curves. The determined validation parameters included the apparent recovery, analytical range, limit of detection, sensitivity and intra-assay accuracy and precision. The fitness for purpose of the screening methods was determined by evaluating the cut-off value and false suspect rate. The cut-off value of the immunoassay method can be defined as the determined response or concentration above which the sample is classified as suspect.

### 3.6. Dust Correlation Study

Using the developed and validated sample preparation and ELISA method, the DON content of the 16 wheat and corresponding wheat dust samples was determined. All calculations were performed and processed using Microsoft Office Excel 2010 (Microsoft Corporation, Redmond, WA, USA) and IBM SPSS 22 (IBM Analytics, Armonk, NY, USA). Verifying a possible correlation, the Pearson correlation coefficient was determined for the batch of samples.

## 4. Conclusions

An aqueous sample preparation and two detection methods were developed for the screening of DON in wheat and wheat dust. For the detection, the use of ELISA was compared to two sensor-based immunoassays namely, SPR and BLI. In buffer, a sevenfold better sensitivity was seen for SPR compared to direct ELISA; however, for wheat and wheat dust extract, a very high matrix effect was observed, making SPR impossible to use for DON screening of aqueous wheat extracts. Similar antibody IC_50_ values were determined for direct ELISA and BLI in buffer, wheat and wheat dust extract. A high affinity was determined between the antibody and the DON-OVA conjugate in ELISA and BLI, making it possible to detect DON-OVA in a pM range in ELISA and in an at least 20 times lower amount in BLI. As a 100fold lower affinity for DON was determined, DON contaminated wheat and wheat dust samples can be analyzed with the antibody in a μM range. Both ELISA and BLI methods were successfully validated according to the European Commission Regulation 519/2014/EC and Commission Decision 2002/657/EC.

The developed ELISA method was used in an experimental field trial, where a linear (*r* = 0.889, *p*-value = 0.044) correlation was found between the DON content in wheat dust *versus* the DON content in wheat. More wheat and wheat dust samples need to be collected and analyzed to further increase the certainty of the obtained linear regression and to further prove the possibility of estimating the DON content in cereals through the determination of DON in dust.

The chemical composition results of wheat dust reveal that certain wheat dust samples can have a very high mineral content. As a high extraction volume (0.5 g dust in 25 mL water) was used, the concentration of these minerals and their influence on the developed immunoassay format can be considered as negligible.

In general, the sampling of dust and subsequent ELISA analysis can be considered as a fast and easy-to-use technique which can be performed on-site. BLI is an automated sensor and can be left unattended during all detection steps after adding the sample extracts. Individual assays took approximately 7.5 min to conduct, making it less time consuming than ELISA. The incorporation of BLI in a rapid biosensor for DON determination on the field looks promising.

## Figures and Tables

**Table 1:** Mineral composition of wheat dust.

Mineral	Concentration Batch 1 (μg/g) (*n* = 2)	Concentration Batch 2 (μg/g) (*n* = 2)
Cu	51	339
Fe	27,400	13,450
K	31,700	79,300
Mg	8700	44,650
Mn	768	2320
Na	1900	11,100
P	10,400	46,150
S	3630	9870
Zn	700	3380

**Table 2:** Direct enzyme-linked immunosorbent assay (ELISA) recovery results for spiked wheat dust.

Spiking Level	Dilution of Extract	DON Concentration Found (μg/kg)	Recovery (%)
**4000 μg/kg**	1/10	2060	52
	1/100	5010	125
	1/1000	52,110	*
**12,000 μg/kg**	1/10	3780	32
	1/100	1980	17
	1/1000	100,950	*
**20,000 μg/kg**	1/10	7340	37
	1/100	4510	23
	1/1000	23,220	116

* Outside the linearity range.

**Table 3:** Overview of validation results of the wheat and wheat dust ELISA screening methods.

Validation Parameters	Wheat			Wheat Dust		
	Spiking Level (μg/kg)	Results		Spiking Level (μg/kg)	Results	
		ELISA	BLI		ELISA	BLI
*r*	-	0.998	0.985	-	0.998	0.991
Apparent recovery	1000	96%	99%	2000	96%	83%
	2000	102%	100%	10,000	108%	105%
LOD	-	233 μg/kg	128 μg/kg	-	458 μg/kg	737 μg/kg
RSD_r_	1500	3.87%	4.00%	2000	7.33%	5.05%
	3000	7.93%	4.11%	8000	9.28%	3.61%
RSD_R_	1500	7.50%	7.30%	2000	8.16%	8.36%
	3000	8.73%	5.58%	8000	9.47%	7.00%
U	-	<22%	<32%	-	<29%	<34%
False suspect result	-	<0.01%	<0.01%	-	<0.01%	<0.01%

*r* = correlation coefficient; LOD = limit of detection; RSD_r_ = intra-day precision; RSD_R_ = inter-day precision; U = expanded measurement uncertainty.

**Table 4:** Summary of immunoassay formats used.

Immunoassay	Format		Determined Characteristic
**ELISA**	Direct	Competitive	IC_50_ in buffer, wheat extract and wheat dust extract
	Indirect	Competitive	IC_50_ in buffer Affinity to DON in buffer
		Non-competitive	Affinity to DON-OVA in buffer, wheat extract and wheat dust extract
**SPR**	Indirect	Competitive	IC_50_ in buffer
		Non-competitive	Affinity to DON-OVA in buffer
**BLI**	Indirect	Competitive	IC_50_ in buffer, wheat extract and wheat dust extract
		Non-competitive	Affinity to DON-OVA in buffer, wheat extract and wheat dust extract

## Abbreviations

The following abbreviations are used in this manuscript

ACGIH	American Conference of Governmental Industrial Hygienists
APS	aminopropylsilane
BLI	biolayer interferometry
CBS	carbonate buffered saline
CDI	*N*,*N*′-carbonyldiimidazole
C-PBS	Casein-PBS
DMF	dimethylformamide
DON	deoxynivalenol
EC	European Commission
EDC	1-ethyl-3-[3-dimethylaminopropyl] carbodiimide
ELISA	enzyme-linked immunosorbent assay
FPIA	fluorescence polarization immunoassay
GC	gas chromatography
Gly	glycine
HEPES	2-[4-(2-hydroxyethyl)piperazin-1-yl]ethanesulfonic acid
HPLC	high performance liquid chromatography
HRP	horseradish peroxidase
IC50	half maximal inhibitory concentration
ICP-AES	atomic emission spectroscopy
IgG	immunoglobulin G
KD	equilibrium dissociation constant
LC-MS/MS	liquid chromatography tandem mass spectrometry
LOD	limit of detection
MS	mass spectrometry
NHS	*N*-hydroxysuccinimide
OVA	ovalbumin
PB	phosphate buffer
PBS	phosphate buffered saline
PBST	phosphate buffered saline Tween 20
*r*	correlation coefficient
RSD	relative standard deviation
RU	response units
SPR	surface plasmon resonance
STC	screening target concentration
TMB	3,3′,5,5′-tetramethylbenzidine
